# Intravenous Artesunate Treatment of Severe Malaria in a Patient With Systemic Lupus Erythematosus: A Case of Post-Artesunate Delayed Hemolysis

**DOI:** 10.7759/cureus.44201

**Published:** 2023-08-27

**Authors:** Enad Alsolami

**Affiliations:** 1 Internal Medicine, College of Medicine, University of Jeddah, Jeddah, SAU; 2 Internal Medicine, Saudi German Hospital, Jeddah, SAU

**Keywords:** parasite, artesunate, hemolysis, anemia, lupus nephritis, malaria

## Abstract

Systemic lupus erythematosus (SLE) is an autoimmune condition linked to multi-organ damage, and its correlation with malaria has been theorized. This case report details a 14-year-old Sudanese girl diagnosed with SLE and severe malaria who experienced hemolytic anemia following intravenous artesunate treatment. Intravenous artesunate was administered as the recommended treatment for severe malaria for one week and led to prolonged hemolysis with low hemoglobin levels and elevated lactate dehydrogenase activity; over three weeks, the hemolysis gradually subsided. This case highlights the need to consider post-artesunate (or artemisinin) delayed hemolysis (PADH) as a potential complication among patients receiving artemisinin derivatives for malaria treatment, thus necessitating enhanced surveillance strategies and further investigation of its mechanisms to optimize clinical practice and patient outcomes in this population.

## Introduction

Systemic lupus erythematosus (SLE) is an autoimmune disorder in which antibodies are produced against self-antigens and form immune complex deposits in tissues, leading to multi-organ damage. Significant morbidity and mortality are associated with lupus nephritis, one of SLE's most severe clinical manifestations. The renal dysfunction rate is higher in the Asian population, and 50-60% of SLE patients suffer renal dysfunction [1].

The 2021 World Malaria Report confirmed a rise in malaria cases from 227 million in 2019 to 241 million in 2020 [2]. Malaria is mostly a globally prevalent disease but is usually found in sub-tropical areas such as Africa and South Asia [3]. The treatment of malaria is usually based on the infecting species, the patient's clinical status, and any past antimalarial medication given. Severe malaria is caused by *Plasmodium falciparum*. It is a significant cause of worldwide morbidity and mortality and is responsible for around 40,000 deaths per year globally, with cases majorly from Africa [3]. It is usually characterized by parasite density >5% (hyperparasitemia), hemoglobin levels <7 g/dL, jaundice, acute kidney problems, and splenomegaly [3]. Malaria has been associated with various complications including liver or renal impairment, cerebral malaria, acute respiratory distress syndrome, and spontaneous splenic rupture [4].

An aggressive treatment for severe malaria is recommended in the form of intravenous (IV) artesunate [1], the parenteral form of artemisinin derivatives. It is extracted from the Chinese medicinal plant *Artemisia annua* [5]. Although not licensed by the United States Food and Drug Administration (FDA), it is considered the first line of defense for treating severe malaria in Africa and Asia [3]. Artesunate became the prioritized option as it reduced the mortality rate of adults significantly in Asia (from 22% to 15%) and for children in Africa (10.9% to 8.5%) [2].

A severe complication of post-artesunate (or artemisinin) delayed hemolysis (PADH) is reported in various incidences [2,5], depending on the severity of malaria, the dose and duration of artesunate, and the time of hemoglobin measurement. The incidence rate can range from 11% to 42% among non-immune travelers with severe malaria who received IV artesunate [2]. PADH complication usually occurs one to three weeks post-artesunate treatment; traditionally seen in endemic areas and non-immune travelers diagnosed with severe malaria [2,5]. The exact mechanism of PADH has yet to be fully understood. One study suggests the direct effect of artesunate on the infected erythrocytes induces delayed hemolysis. It also associated malarial parasitic density with the degree of hemolysis [6].

The current case describes the complication of PADH in a patient who was treated for severe malaria with artesunate that resulted in the development of hemolytic anemia in the patient post artesunate treatment.

## Case presentation

A 14-year-old Sudanese girl presented to the Nephrology clinic of our hospital with a fever. The patient had moved from Sudan to Saudi Arabia a month prior to the presentation. The patient’s medical history included class 3 and 4 lupus nephritis, hypertension, and chronic kidney disease. Lupus nephritis was diagnosed in Sudan, two months prior to her presentation. She had been treated with methylprednisolone, tacrolimus, and mycophenolate mofetil for lupus nephritis, and also received three sessions of hemodialysis at that time. She was on three anti-hypertensive medications, including amlodipine (10 mg) once daily, indapamide (1.5 mg) once daily, and hydralazine (50 mg) three times a day. The biochemistry profile of the patient at the time of presentation is represented in Table 1.

**Table 1:** The biochemistry profiles of the patient at presentation

	Levels in the patient at presentation	Normal reference range
Serum creatinine	3 mg/dl	0.57-1.1 mg/dl
Proteinuria	3+/hpf	Negative
Red blood cells	2+/hpf	Negative
Hemoglobin	8 g/dL	12-16 g/dl
Platelet counts	211 x 109 /L	150-400 x 109/L
Anti-double strand DNA	Normal	Normal
Complements 3 and 4	Normal	Normal

On admission to the hospital, broad-spectrum antibiotics were initiated for the treatment of possible bacterial infection in the immunocompromised patient. Further laboratory examination revealed a high lactate dehydrogenase (LDH) with 444 U/L, haptoglobin level was undetectable on day one and the direct Coombs test was negative. Normal values of LDH and haptoglobin levels are 140-280 LDH units/L and 40-200 mg/dL, thus indicating non-immune hemolytic anemia. The blood film was positive for *Plasmodium falciparum* (>25%). Hence, the patient was also confirmed for severe malaria based on the clinical presentation and laboratory investigations.

She was treated with IV artesunate from day one of hospital admission for one week and received multiple blood transfusions. Her fever subsided after two days of treatment initiation. She developed hemolytic anemia on day one of admission, which was attributed to severe malaria. Blood film was negative for *Plasmodium falciparum* on day three after starting IV artesunate. However, hemolysis perpetuated even after the malaria film tested negative, indicating a persistent hemolytic condition due to artesunate treatment. On the seventh day after hospital admission, the patient developed chronic kidney disease (CKD), and hemodialysis was started.

**Figure 1:**
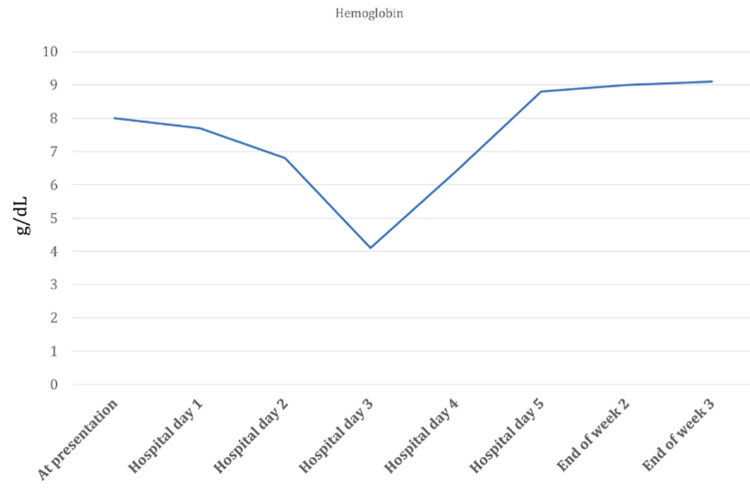
Hemoglobin levels from the time of hospital admission till the third week post artesunate treatment.

**Figure 2:**
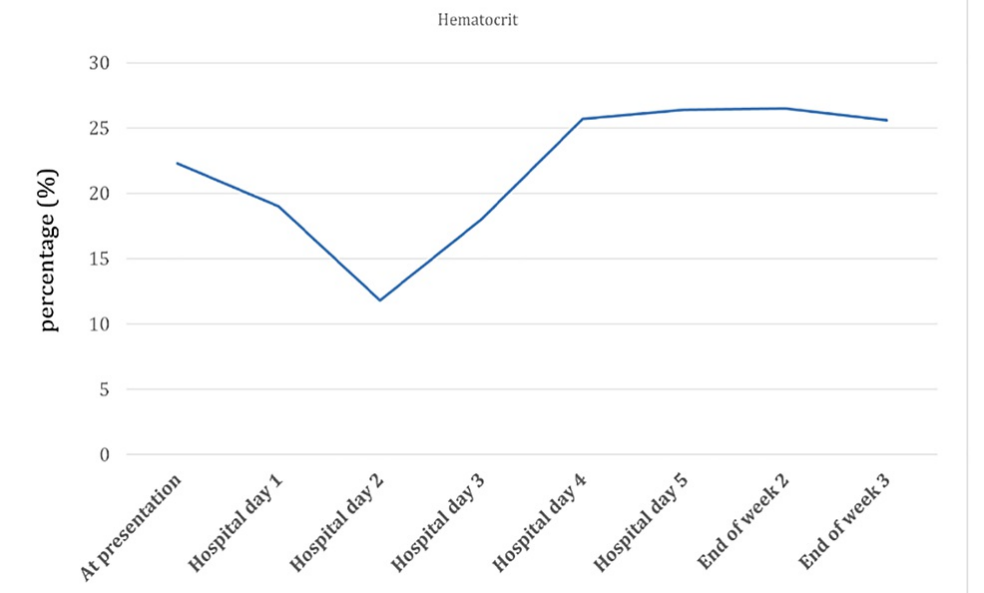
Hematocrit levels from the time of hospital admission till three weeks post artesunate treatment.

**Figure 3:**
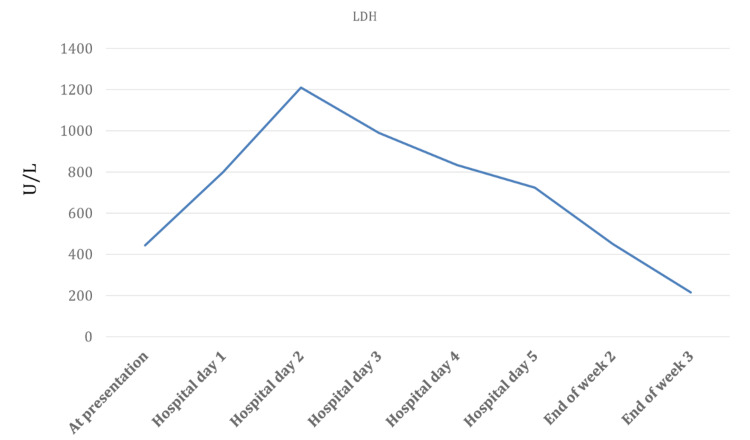
Lactate dehydrogenase (LDH) levels from the time of hospital admission till three weeks post artesunate treatment.

On the second day after hospitalization, her hematocrit and LDH levels (Figures 1-2) were at the lowest (11.8%) and highest (1210 units/L), respectively. Normal values for hematocrit and LDH levels are 40-50% and 140-280 units/L. Her hemoglobin level was lowest on the third day after hospitalization and IV artesunate treatment (4.1 g/dL) (Figure 3). Haptoglobin was undetectable (<7.6 mg/dl) two days after initiation of artesunate. Hemolysis persisted in the background till the end of week two, as haptoglobin remained undetectable despite improvement in LDH and hemoglobin levels.

Observed outcomes

The patient’s hemoglobin, hematocrit, and LDH levels gradually improved; the haptoglobin levels too improved, and an average level of haptoglobin was achieved by the end of the second week after hospitalization (Table 2). Even after three weeks of IV artesunate treatment, she continued to have anemia. This was likely due to CKD.

**Table 2:** Hemoglobin, hematocrit, LDH, and haptoglobin levels through the IV artesunate treatment. LDH: lactate dehydrogenase

		Hemoglobin (g/dL)	Hematocrit (%)	LDH (Units/L)	Haptoglobin (mg/dl)
Normal Reference Range		(12-16)	(40-50)	(140-280)	(40-200)
At presentation		8	22.3	444	--
Week 1	Hospital day 1	7.7	19	799	<7.6
	Hospital day 2	6.8	11.8	1210	<7.6
	Hospital day 3	4.1	18	990	<7.6
	Hospital day 4	6.4	25.7	834	<7.6
	Hospital day 5	8.8	26.4	724	<7.6
Week 2	Mid 2^nd^ week	8.6	24.1	--	<7.6
	End of week 2	9	26.5	450	37.5
Week 3	End of week 3	9.1	25.6	215	39

## Discussion

This case report highlights an unusual incident of prolonged hemolysis following IV artesunate treatment for severe malaria. Clinical observations revealed an improvement in her condition over weeks of hospitalization. In this case, malaria was diagnosed promptly, emphasizing the importance of suspecting malaria infection by performing malaria antigen testing and blood smears on any patient who becomes ill in a malaria-endemic area [7]. An IV artesunate dose could cause delayed extravascular hemolysis between seven and 21 days after treatment initiation in those with hyperparasitemia [8]. PADH is associated with a drop in hemoglobin and subsequent rise in lactate dehydrogenase (LD), but no sign of recurrent parasitemia.

Hemolytic anemia can have various potential causes that need to be considered for accurate diagnosis. These include autoimmune hemolytic anemia, genetic hemoglobinopathies such as sickle cell disease and thalassemia, enzyme deficiencies such as glucose-6-phosphate dehydrogenase (G6PD) deficiency, conditions like hereditary spherocytosis and paroxysmal nocturnal hemoglobinuria, microangiopathic disorders like thrombotic thrombocytopenic purpura and hemolytic uremic syndrome, infections like malaria, drug-induced reactions, and mechanical factors like heart valve defects [9,10]. A thorough evaluation is crucial to differentiate and identify the specific underlying cause [9,10]. Complications associated with artesunate may occur in up to 7-23% of severe malaria cases requiring this medication and are generally self-limiting with no reported long-term sequelae. Patients usually recover with supportive therapy but may require a transfusion of blood products if anemia becomes severe. Hyperparasitemia (parasite load between 4-37%) appears to be a risk factor for PADH [8,11].

As evidenced by the 2021 World Malaria Report, malaria cases continue to surge globally, underscoring the significance of understanding any potential complications related to its treatment. PADH incidents demonstrate the need to take extra precautions when prescribing artesunate to those suffering from SLE and related autoimmune conditions and other autoimmune-mediated illnesses [12,13]. This case adds to the existing literature by providing insight into the risk of prolonged hemolysis among patients taking artesunate for severe malaria. These findings warrant further exploration into its underlying mechanisms and consider treatment options to optimize future clinical practice and patient outcomes.

Artesunate, an IV artemisinin derivative extracted from *Artemisia annua*, is often prescribed as the first-line treatment for severe malaria [1-3]. Unfortunately, PADH is an adverse event typically occurring one to three weeks post treatment in areas of high prevalence and non-immune travelers diagnosed with severe malaria [2,5].

This case emphasizes the significance of considering PADH as a potential complication in patients who take artemisinin derivatives for malaria treatment, adding further evidence that such hemolysis occurs in both endemic areas and non-immune travelers with severe malaria. While its exact mechanism remains unknown, one study suggests artesunate may directly target infected erythrocytes leading to delayed hemolysis [14].

This case presents an unusual finding, PADH in a patient with SLE, underscoring the need for close monitoring and awareness of this potential complication. Understanding risk factors and mechanisms causing PADH will assist clinicians in timely recognizing and managing any adverse event. Future clinical practice could benefit from this evidence by including enhanced surveillance strategies explicitly tailored for patients who require artemisinin derivatives to treat malaria, improving both outcomes and safety while improving patient outcomes and safety.

## Conclusions

The current case report informed about PADH in severe malaria patients with lupus nephritis. PADH complications emerged after three weeks of IV artesunate treatment, with the exact mechanism is unknown. This case gives insight into PADH as a potential complication in patients with SLE under artemisinin derivatives therapy for malaria. PADH is a rare event post artesunate treatment with fatal complications warranting close monitoring and assessment. To prevent complications and ensure a positive outcome for the patient, PADH must be recognized and treated promptly. Clinicians should consider risk factor analysis and explore mechanisms of PADH. Enhanced surveillance strategies for patients with malaria detection will ensure better safety outcomes.
